# STYK1 promotes epithelial-mesenchymal transition and tumor metastasis in human hepatocellular carcinoma through MEK/ERK and PI3K/AKT signaling

**DOI:** 10.1038/srep33205

**Published:** 2016-09-15

**Authors:** Zhaowen Wang, Lei Qu, Biao Deng, Xing Sun, Shaohan Wu, Jianhua Liao, Junwei Fan, Zhihai Peng

**Affiliations:** 1 Department of General Surgery, Shanghai General Hospital, Shanghai Jiao Tong University, 100 Haining Road, Shanghai, 200080, China

## Abstract

Serine/threonine/tyrosine kinase 1 (STYK1) is known to be involved in tumor progression. However, its molecular role and mechanism in hepatocellular carcinoma (HCC) remains unknown. We evaluated the effect of STYK1 expression in HCC tissues and investigated the underlying mechanisms associated with progression. HCC tissues expressed greater levels of STYK1 than paired non-tumor tissues. Patients with HCC expressing low levels of STYK1 showed both, greater disease-free (p < 0.0001) and overall (p = 0.0004) survival than those expressing high levels of STYK1. Decreased expression of STYK1 was significantly associated with decreased cell proliferation, reduced migratory capability, and reduced invasive capability. Overexpression of STYK1 was significantly associated with increased cell proliferation, migratory capability, and invasive capability *in vitro*, as well as increased volume of tumor, weight of tumor, and number of pulmonary metastases *in vivo*. Furthermore, STYK1’s mechanism of promoting cancer cell mobility and epithelial-mesenchymal transition (EMT) was found to be via the MEK/ERK and PI3K/AKT pathways, resulting in increased expression of mesenchymal protein markers: snail, fibronectin, and vimentin, and decreased E-cadherin expression. Our results suggest that STYK1 acts as an oncogene by inducing cell invasion and EMT via the MEK/ERK and PI3K/AKT signaling pathways and it therefore may be a potential therapeutic target in HCC.

Hepatocellular carcinoma (HCC) is the second most common form of cancer causing death. HCC has been associated with an annual incidence of over half a million individuals, globally^1^,^2^, which continues to rise in spite of preventative measures. As hepatitis B and C-related liver cirrhosis is the most common risk factor, HCC is commonly seen in patients of East Asian and African descent^2^. However, alcoholic cirrhosis and increasingly nonalcoholic steatohepatitis (NASH) are additional important risk factors that may possibly add to the incidence in the Western world as well, in the near future^1^. Although surgical techniques and adjuvant therapy have improved, long-term survival remains low due to metastasis^3^. Aberrant activation and dysfunction of crucial genes result in progression of HCC^4^,^5^,^6^. Therefore, it is critical to identify specific molecular markers and elucidate the molecular mechanisms involved in HCC that could serve as clinical/prognostic factors.

Serine/threonine/tyrosine kinase 1 (also known as NOK), a human putative protein kinase, belongs to the receptor protein tyrosine kinase family and comprises a transmembrane domain and an intracellular domain^7^,^8^. Upregulation of STYK1 expression has been implicated in many types of tumors, including breast cancer^9^,^10^, non-small cell lung cancer^11^, ovarian cancer^12^, and colorectal cancer^13^. Most previous studies focused on the expression of STYK1 in cancer tissues. However, the expression and role of STYK1 in HCC remains unknown. In addition, STYK1 is overexpressed in castration-resistant prostate cancer cells and tissues, and enforced expression of STYK1 promoted cell proliferation *in vitro*^14^. Furthermore, STYK1 promotes cell transformation, tumorigenesis, and metastasis by activating of phosphoinositide 3-kinase (PI3K)/AKT and inactivating GSK-3β signaling pathway, which are implicated in tumorigenesis and development of HCC^7^,^15^.

Accumulating evidence has shown that epithelial-to-mesenchymal transition (EMT), characterized by the progression of epithelial phenotype to mesenchymal phenotype, plays a critical role in cancer invasion and metastasis during tumor progression^16^,^17^,^18^. EMT-associated genes are reported to be associated with HCC recurrence and metastasis^19^,^20^,^21^. In HCC, EMT results in increased venous invasion, metastasis, and a poorer prognosis^5^,^20^,^21^. Despite evidence of STYK1 acting as an oncogene and being involved in a variety of cancers, the role of STYK1 in regulating HCC metastasis and EMT remains unclear.

In this study, we estimated STYK1 expression and its effects on invasion and metastasis of HCC. We also investigated the precise mechanism/pathway by which STYK1 contributes to invasion and EMT events *in vitro* and *in vivo*. These findings may provide a potential therapeutic target to inhibit HCC progression and metastasis.

## Results

### STYK1 levels are elevated in HCC tissue compared to paired non-tumor tissue

**Figure 1:**
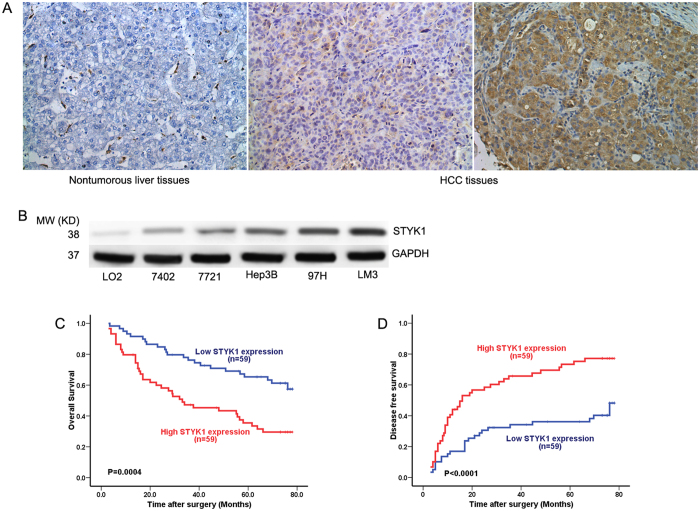
STYK1 expression in HCC. (**A**) Representative immunohistochemical staining for STYK1 in HCC and paired, non-tumor hepatic tissues revealed greater amounts of STYK1 in HCC tissue (original magnification, 200×). (**B**) Western blot assays performed on HCC cell lines as indicated. (**C**) Kaplan-Meier analysis revealed a statistically significant correlation between tumors that express high levels of STYK1 and reduced overall survival. (**D**) Kaplan-Meier analysis reveals a statistically significant correlation between tumors that express high levels of STYK1 and reduced DFS.

Immunohistochemical staining revealed the presence of STYK1 at significant levels in HCC tissues and relatively insignificant levels in paired, non-tumor tissues (Fig. 1A). Then we detected the expression of STYK1 in human normal liver cell line LO2 and 5 HCC cell lines (7402, 7721, Hep3B, 97H, and LM3), and results demonstrated a higher expression of STYK1 in HCC cell lines, compared with LO2 (Fig. 1B). These results suggested that STYK1 might play an important role in HCC progression and development.

### STYK1 level functions as an independent predictor of survival in patients with HCC

To further explore the potential role of STYK1 in HCC progress, we evaluated STYK1 expression and the clinicopathological characteristics are summarized in Table 1. The patients were divided to two groups (low STYK1 and high STYK1) according to median of STYK1 expression in cancer tissues samples. STYK1 overexpression was significantly correlated with tumor size, vascular invasion, and a higher tumor-nodule-metastasis (TNM) stage. Patients with HCC tumors expressing low STYK1 levels had significantly higher overall survival (OS) than those expressing high STYK1 levels (p = 0.0004) (Fig. 1C). Similarly, patients with HCC tumors expressing low STYK1 levels showed significantly longer disease-free survival (DFS) than those expressing high STYK1 levels (p < 0.0001) (Fig. 1D). Moreover, tumor size, tumor number, vascular invasion, and advanced TNM stage were found associated with lower OS and shortened time-to-recurrence (TTR) in univariate analysis (Table 2). Multivariate Cox regression analysis further revealed that STYK1 was an independent risk factor for lower OS and shortened TTR in patients with HCC (Table 3).

### STYK1 silencing inhibits HCC cell growth, migration, and invasion

**Figure 2:**
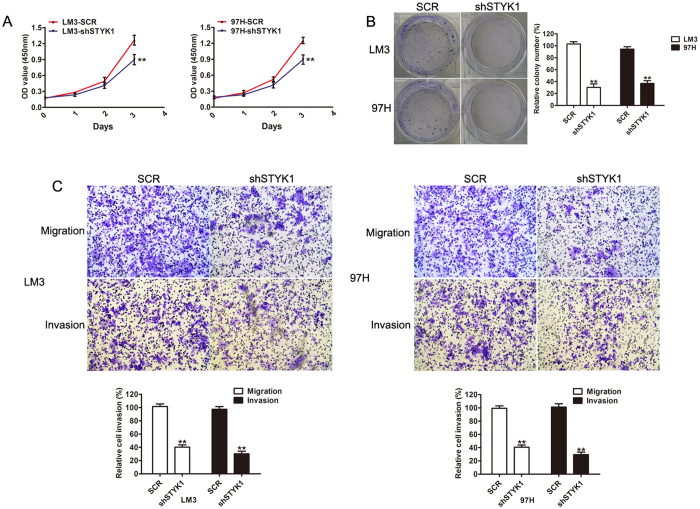
Reduction of STYK1 expression in HCC cells via shSTYK1 inhibits cell proliferation, migration, and invasion. (**A**) LM3 and 97H cells with shSTYK1 exhibited significantly lower rates of cell proliferation. (**B**) Representative images of colony formation by LM3 and 97H cells with shRNA-scramble or shRNA-STYK1, demonstrating decreased colony formation in cells treated with shRNA-STYK1. (**C**) Representative images of transwell assays performed with LM3 and 97H cells with shRNA-scramble or shRNA-STYK1, demonstrating reduced migratory and invasive capacity (original magnification, 100×).

To explore the role of STYK1 in the progression of HCC, short hairpin RNA (shRNA) were used to silence endogenous expression of 2 HCC cell lines: LM3 and 97H, which show high malignant and metastatic capacity. Western blot was used to confirm decreased expression of STYK1 in LM3 and 97H cell lines following transfection with negative vector (SCR) and different knockdown vectors. Sh3, targeting STYK1, exhibited significantly decreased expression of STYK1 as compared to SCR, sh1, and sh2; therefore it was selected for subsequent studies (Supplementary Fig. 1). MTT assay revealed that the proliferation rate of LM3 and 97H cells transfected with STYK1-knockdown was lower than that of negative vector cells (Fig. 2A). A colony-formation assay was further performed to assess cell proliferation. Similarly, LM3 and 97H cells with STYK1 silencing exhibited significantly smaller and lower number of colonies (Fig. 2B). In addition, migratory and invasive capacities were assessed using a transwell assay. As shown in Fig. 2C, inhibition of STYK1 expression resulted in decreased cell migration and invasion in both, LM3 and 97H cells. In summary, our results indicate that down-regulation of STYK1 decreased the proliferation and metastatic ability of HCC *in vitro*.

### Overexpression of STYK1 promotes HCC cell growth *in vitro* and *in vivo*

**Figure 3:**
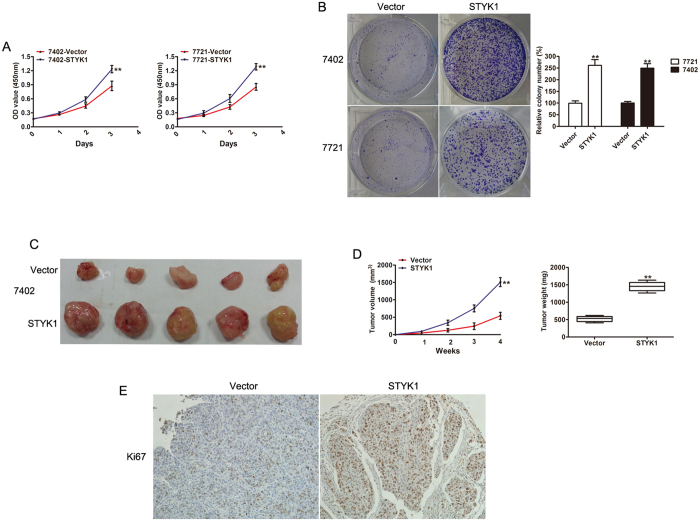
Overexpression of STYK1 promotes HCC cell proliferation and tumor growth both *in vitro* and *in vivo*. (**A**) MTT assay revealed increased cell proliferation of BEL-7402 and SMMC-7721 cells with STYK1 compared to control. (**B**) Representative images of colony formation of BEL-7402 and SMMC-7721 cells with control vector or STYK1, demonstrating increased colony formation in cells with the STYK1. (**C**) Representative images of tumor at sacrifice, obtained from mice implanted with BEL-7402 HCC cells with either empty vector or STYK1. (**D**) Tumor volume and tumor weight curves, demonstrating that tumors derived from BEL-7402 cells with STYK1 vector have significantly greater volumes and weights after 4 weeks than those derived from HCC cells with control vector. (**E**) Reprehensive IHC staining for Ki67 expression in xenograft tumor tissues from the control vector or STYK1 vector. **P  <  0.01.

To further investigate the effects of STYK1 on HCC cells, we overexpressed STYK1 in 7721 and 7402 HCC cells by lentiviral transduction. Western blot was used to confirm the increased levels of STYK1 in cells transfected with STYK1 vector (Supplementary Fig. 2). Ectopic STYK1 expression significantly promoted proliferation activity in both, 7721 and 7402 cells (Fig. 3A). In addition, compared with control vector, the colony-formation assay showed that the size and number of colonies formed by 7721 and 7402 cells were significantly increased by stable STYK1 expression (Fig. 3B). Furthermore, to assess the effects of STYK1 on HCC growth *in vivo*, 7402 cells with control vector or STYK1 were injected subcutaneously into nude mice. The tumors size was significantly larger in the STYK1-expression group compared with the control group (Fig. 3C). The growth curve and weight of dissected tumor further confirmed the progress of tumor cell growth caused by STYK1 overexpression (Fig. 3D). IHC also indicated more Ki67 antigen-positive cells in tumors derived from 7402 cells with STYK1 compared with tumors derived from vector (Fig. 3E). These data indicate that STYK1 played the role of an oncogene in HCC progression.

### **Overexpression of STYK1 promotes cell migration and invasion** ***in vitro*** **and** ***in vivo***

**Figure 4:**
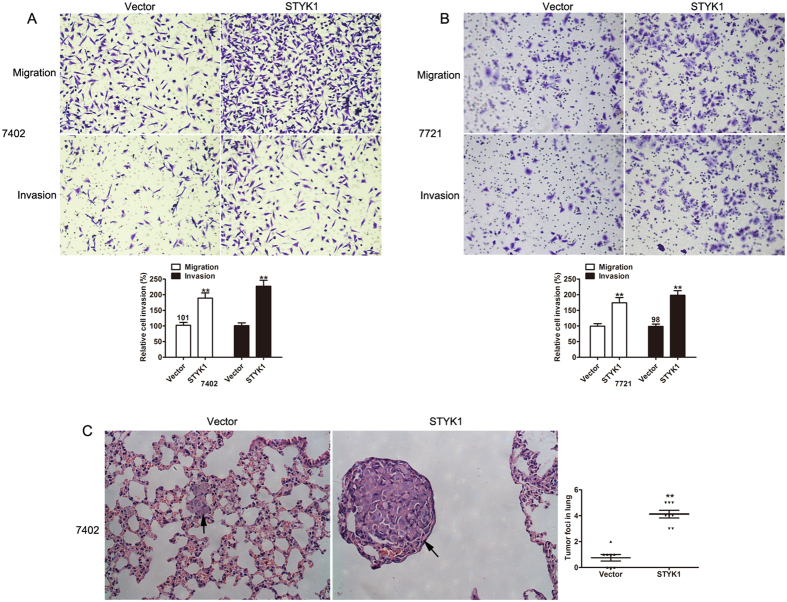
Overexpression of STYK1 increases HCC cell migratory and invasive capacity *in vitro* and number of pulmonary metastases *in vivo*. (**A**) BEL-7402 HCC cells with STYK1 have significantly increased migratory and invasive capacity compared to cells with control vector. (**B**) SMMC-7721 HCC cells with STYK1 have significantly increased migratory and invasive capacity compared to cells with control vector. (**C**) Representative histopathologic images of the lungs demonstrating metastatic nodules are shown in the left panel. Mice implanted with BEL-7402 cells with STYK1 had a significantly larger number of pulmonary metastases than mice implanted with HCC cells with the control vector, as is summarized in the right panel. **P  <  0.01.

The invasive and migratory capacities of HCC cells with STYK1 vector were assessed using a transwell assay. As expected, ectopically expressed STYK1 in 7721 and 7402 cells greatly increased migration and invasion abilities, compared with those shown by control vector cells (Fig. 4A,B). We then investigated if STYK1 enhanced cancer cell metastasis *in vivo*. As shown in Fig. 4C, ectopic expression of STYK1 resulted in increased metastatic foci and larger nodules in the lung. The results indicate that overexpression of STYK1 in 7402 cells led to significant promotion of cell invasion and lung metastasis *in vitro* and *in vivo*.

### STYK1 induces epithelial-mesenchymal transition in HCC cells

**Figure 5:**
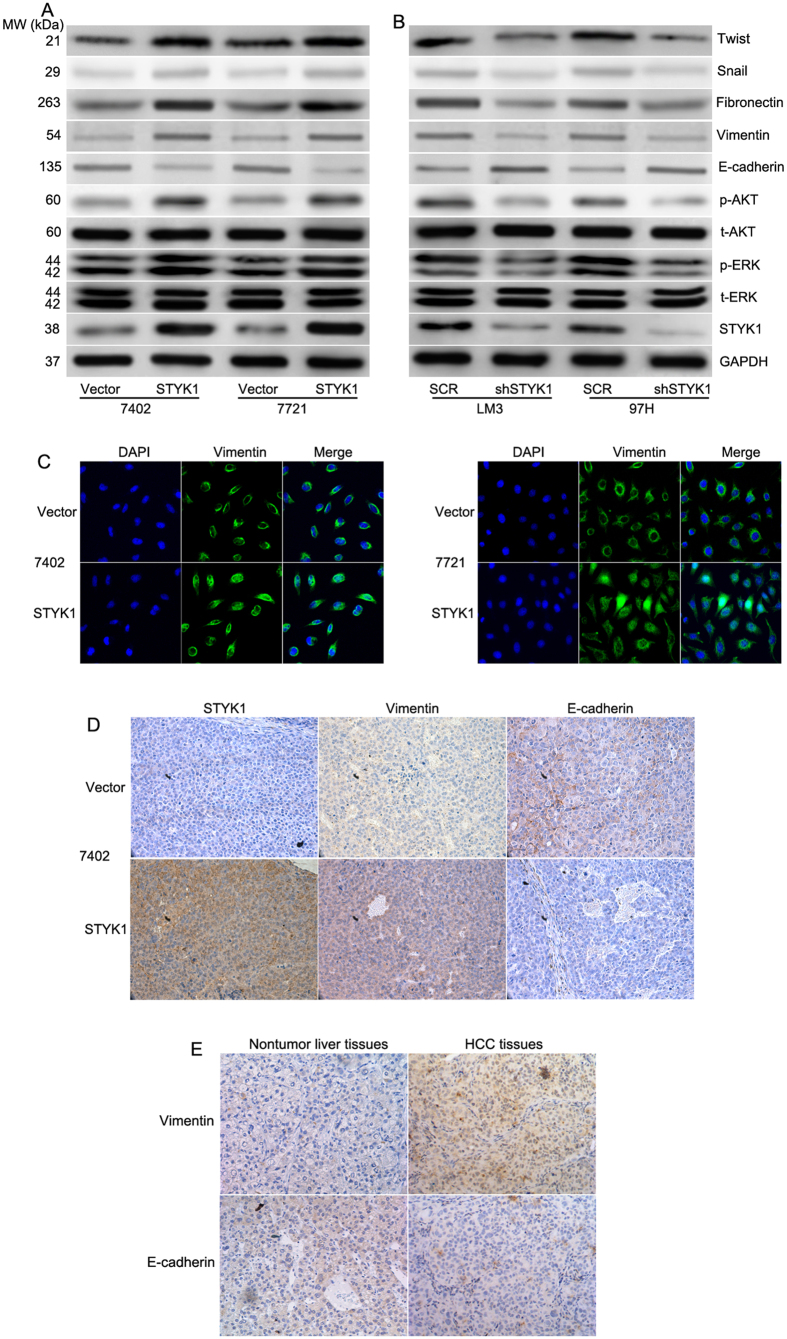
STYK1 activates the MEK/ERK and PI3K/AKT pathways to induce EMT. (**A**) Relative expression levels of STYK1, ERK, phosphorylated ERK, AKT, phosphorylated AKT, E-cadherin, vimentin, fibronectin, and Snail in BEL-7402 and SMMC-7721 HCC cells with control vector or STYK1, respectively. (**B**) Relative expression levels of STYK1, ERK, phosphorylated ERK, AKT, phosphorylated AKT, E-cadherin, vimentin, fibronectin, and Snail in LM3 and 97H HCC cells with shRNA-scramble or shRNA-STYK1, respectively. (**C**) Immunofluorescence images with staining for the mesenchymal marker vimentin of BEL-7402 and SMMC-7721 cells with either control vector or STYK1 demonstrated increased expression of vimentin in those cells with STYK1. (**D**) Immunohistochemical staining of the paraffin tissue sections from mice bearing BEL-7402 xenograft tumors demonstrated increased levels of STYK1 accompany with the upregulation of EMT marker vimentin and downregulation of E-cadherin *in vivo*. (E) Representative images showing E-cadherin and vimentin expression in in HCC and paired, non-tumor hepatic tissues. **P < 0.01.

Previous studies showed that cell migration and invasion are associated with altered levels of EMT biomarkers^17^. To determine the underlying mechanisms of STYK1 in EMT, western blot analysis was performed to detect the expression of epithelial and mesenchymal protein markers. Our results showed that STYK1 up-regulated the expression of mesenchymal marker proteins: vimentin, fibronectin, twist, and snail while it down-regulated epithelial marker, E-cadherin (Fig. 5A). Conversely, STYK1-depleted LM3 and 97H cells exhibited increased E-cadherin expression, but decreased vimentin, fibronectin, and snail expression (Fig. 5B). MEK/ERK and PI3K/AKT signaling pathway are known to play critical roles in tumor invasion and metastasis. We estimated the alteration of ERK and AKT. As shown in Fig. 5A, overexpression of STYK1 promoted phosphorylation of ERK and AKT, while silencing STYK1 reversed this phenotype (Fig. 5B). To determine whether STYK1 promoted the invasiveness of HCC via EMT, immunofluorescent staining was performed. As shown in Fig. 5C, STYK1 induced vimentin staining in both, 7721 and 7402 HCC cells. To confirm these findings *in vivo*, immunohistochemical analysis was used to determine the expression of epithelial and mesenchymal protein. Consistently, we found that STYK1 overexpression displayed a decrease in the E-cadherin level but an increase in vimentin level (Fig. 5D), which represents the EMT phenotype in the mice bearing the 7402 xenografts. To further confirm the data, the expression of E-cadherin and vimentin were detected by IHC in the same HCC tissues samples. The result showed that E-cadherin was decreased, but vimentin was increased in HCC tissues (Fig. 5E), which consisted with the *in vitro* results. Overall, these results suggest that STYK1 promoted tumor invasion and metastasis by inducing EMT.

### STYK1 mediated epithelial mesenchymal transition in HCC cells occurs via the MEK/ERK and PI3K/AKT signaling pathways

**Figure 6:**
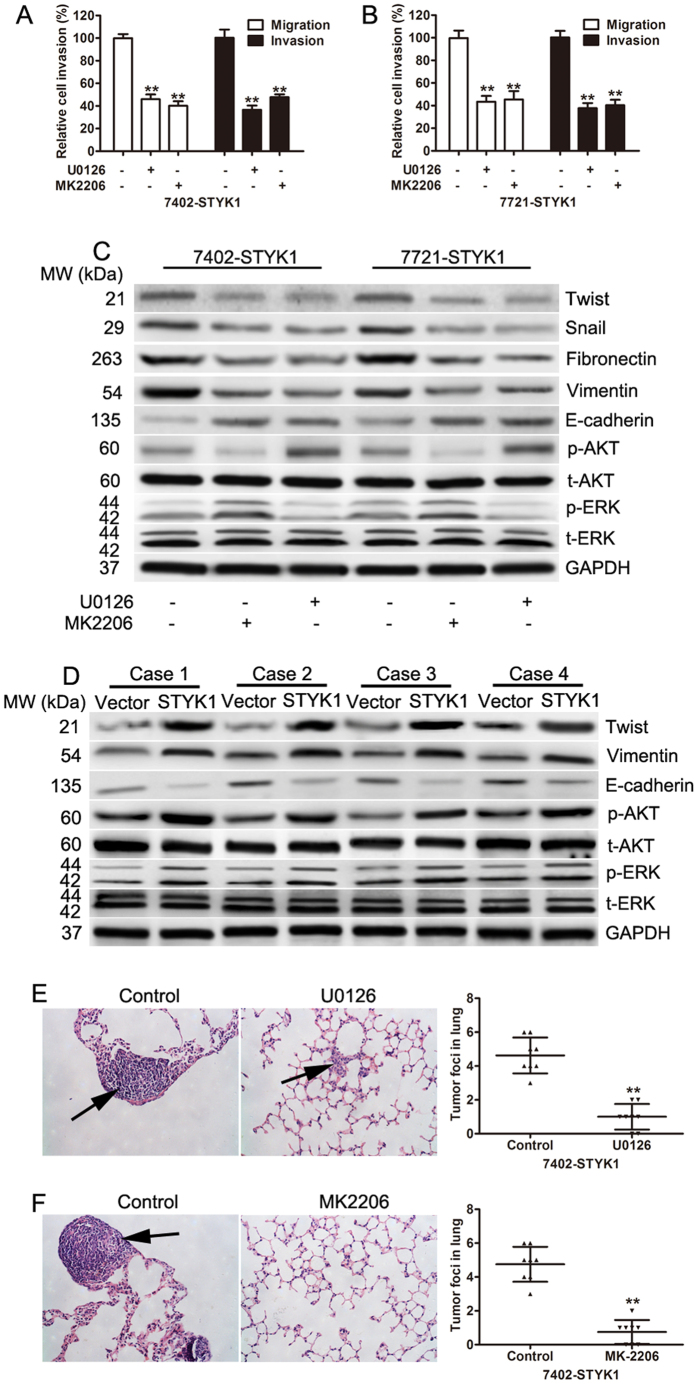
Inhibition of ERK1/2 or AKT reverses the pro-malignant effects of STYK1 overexpression. (**A**) BEL-7402 cells overexpressing STYK1 were treated with the ERK inhibitor U0126 (10 μM) or the AKT inhibitor MEK2206 (25 μM) for 30 minutes, and then the transwell assay was performed 24 h later. Cells that received either U0126 or MEK2206 demonstrated significantly reduced migratory and invasive capacity. (**B**) SMMC-7721 cells overexpressing STYK1 were treated with the ERK inhibitor U0126 (10 μM) or the AKT inhibitor MEK2206 (25 μM) for 30 minutes, and then the transwell assay was performed 24 h later. Cells that received either U0126 or MEK2206 demonstrated significantly reduced migratory and invasive capacity. (**C**) Western blots of BEL-7402 and SMMC-7721 cells overexpressing STYK1 and treated with either ERK inhibitor U0126 or AKT inhibitor MEK2206, or untreated. The results demonstrated a reduction in levels of EMT protein markers and an increase in E-cadherin in cells treated with either the ERK inhibitor or the AKT inhibitor. (**D**) The expression of ERK, phosphorylated ERK, AKT, phosphorylated AKT, E-cadherin, vimentin, and twist was determined in xenograft tumor tissues from the control vector or STYK1 vector. (**E,F**) Representative images of H&E stained mice lung sections from nude mice with 7402 cells harboring STYK1 overexpression treated with U0126 or MK2206. **P < 0.01.

MEK/ERK and PI3K/AKT signaling pathways play important roles in migration, invasion, and metastasis of cancer by regulating EMT; therefore, we investigated if STYK1 was capable of promoting migration, invasion, and EMT via the MEK/ERK and PI3K/AKT pathway in HCC cells. To evaluate whether the effects of STYK1 on cell migration and invasion were dependent on the ERK pathway, 7724 and 7402 cells with stably overexpressed STYK1, were treated with U0126 (ERK inhibitor) or MK2206 (a novel, allosteric AKT inhibitor) for 30 min. Inhibition of ERK or AKT exhibited significantly lower capacities of migration and invasion (Fig. 6A,B). In addition, inhibition of ERK1/2 by U0126 or of AKT by MK2206 reversed the effects on vimentin, fibronectin, twist, and snail up-regulation and E-cadherin down-regulation induced by STYK1 (Fig. 6C). To assess whether regulation of the PI3K/AKT and MEK/ERK signaling by STYK1 could be recapitulated *in vivo*, the tissue lysates of tumors from 4 mice were used to determine the expression of AKT, p-AKT, ERK, p-ERK, E-cadherin, vimentin, and twist by western blotting. Our data suggested overexpression of STYK1 increased the expression of p-AKT, p-ERK, vimentin and twist and decreased the expression of E-cadherin (Fig. 6D). In addition, treatment of nude mice with U0126 (inhibitors of MEK1/2) or MK2206 (inhibitors of AKT) significantly reduced the metastatic nodules induced by STYK1in lung (Fig. 6E,F). These results indicate that STYK1 may induce cell migration, invasion, and EMT through MEK/ERK and PI3K/AKT signaling pathways.

## Discussion

A growing body of evidence has identified genes specifically upregulated or downregulated in HCC tissues that can be considered as early diagnostic markers, prognostic markers, and therapeutic targets^22^,^23^,^24^,^25^. STYK1 is one such a gene that is highly up-regulated in various types of tumors^9^,^10^,^11^,^12^,^14^; however, little is known about its expression in HCC, with even less clarity on its underlying mechanisms. In our present study we found that STYK1 expression was significantly upregulated in HCC tissues. Further correlation analyses suggested that STYK1 overexpression correlated with tumor size, vascular invasion, and higher TNM stage. A multivariable Cox’s regression analysis indicated that STYK1 was a significant risk factor of OS and DFS. In addition, depletion of endogenous STYK1 expression in LM3 and 97H cells suppressed cell growth, migration, and invasion, whereas overexpression of STYK1 in 7721 and 7402 cells had the opposite effect. Moreover, we found that STYK1 induced EMT via the MEK/ERK and PI3K/AKT pathway, which may be responsible for its invasion-promoting activity.

Uncontrolled growth and metastasis, which are important features of malignant tumors, are the main cause of cancer-related death. STYK1 has been reported to promote BaF3 cell proliferation and invasion *in vitro* and *in vivo*^7^. Meanwhile, knockdown of STYK1 decreased proliferation of prostate cancer cell viability *in vitro*^14^.

Here, we demonstrated that STYK1 knockdown inhibited cell proliferation and invasion, while overexpression of STYK1 promoted this effect both *in vitro* and *in vivo*, suggesting the oncogenic role of STYK1 in HCC, consistent with its role in other cancers^7^,^13^. Accumulated data show that EMT results in increasing cell migration and invasion in several cancers^26^,^27^. We found that STYK1 overexpression induced EMT by elevating expression of the mesenchymal markers, vimentin, fibronectin, and snail and by reducing expression of the epithelial marker, E-cadherin. In contrast, increased expression of E-cadherin accompanied by decreased expression of vimentin, fibronectin, and snail were observed in LM3 and 97H with depleted STYK1.

Previous reports show that activation of MEK/ERK and PI3K/AKT contributes to cell growth^28^,^29^, promotes invasion, and EMT^30^,^31^. We showed that phosphorylated levels of ERK and AKT were enhanced by STYK1 overexpression and inhibited by STYK1 silencing, which is consistent with previous reports^7^. Furthermore, we investigated if MEK/ERK and PI3K/AKT signaling are responsible for STYK1-mediated acceleration of tumor cell migration, invasion, and EMT. Blocking MEK/ERK and PI3K/AKT pathway using special inhibitors significantly attenuated STYK1-enhanced migration and invasion of HCC cells. More importantly, U0126 or MEK2206 treatment inhibited the activation of vimentin, fibronectin, and snail as well as inactivation of E-cadherin by STYK1. Inhibition of MEK/ERK and PI3K/AKT pathway with inhibitors significantly reduced the metastatic nodules induced by STYK1 in the lung. Therefore, our data support that activation of MEK/ERK and PI3K/AKT signaling pathways were required for STYK1-stimulated cell migration, invasion, and EMT of HCC cells. STYK1 was previously shown to cause GSK-3β (Ser9) phosphorylation via activating AKT phosphorylation at the Thr308 residue in cervical cancer Hela cell^15^. Several studies have shown aberrant activation of AKT and GSK-3β involved in HCC tumorigenesis, EMT and Metastasis^32^,^33^. The role of STYK1/AKT/GSK3 axis in HCC progress might extend the key functional pathways to abnormal proliferation invasion, and EMT of HCC cells. Transforming growth factor β (TGFβ), a potent inducer of the EMT, have been involved in medicating actin filament reorganization and invasiveness through activating many key genes and signaling pathways, the relation among TGFβ, STYK1 and EMT needed to be further investigated.

In conclusion, we demonstrated that STYK1 is highly expressed in tumor tissues and is significantly correlated with poor outcome of HCC. In addition, STYK1 promoted proliferation, invasion, and metastasis *in vitro* and *in vivo*. We also emphasized that MEK/ERK and PI3K/AKT are required for STYK1-mediated HCC cell invasion and EMT.

## Materials and Methods

### Tissue samples

Paraffin-embedded tumor specimens including HCC tissues and paired non-tumor tissues were obtained from 118 patients with HCC, who underwent surgical resection without prior adjuvant therapy between March 2006 and October 2008 at the Shanghai General Hospital of Shanghai Jiao Tong University. Diagnosis of HCC, tumor differentiation, and presence of lymph node metastases were determined by hematoxylin and eosin (H&E) staining according to the World Health Organization (WHO) classification guidelines (2004). Written consent was obtained from all enrolled patients. This study was approved by the Medical Ethics Committee at Shanghai General Hospital of Shanghai Jiao Tong University. All procedures were carried out in accordance with the Declaration of Helsinki.

### Cell culture

The human HCC cell lines (SMMC-7721 [7721], BEL-7402 [7402], Hep3B, MHCC-97H [97H], and MHCC-LM3 [LM3]) and normal non-malignant liver cells (LO2) were purchased from the Shanghai Cell Bank of the Chinese Academy of Sciences (Shanghai, China) and cultured as our previously described^34^. The MHCC-97H (97H) and HCC-LM3 (LM3) cell lines were acquired from the Liver Cancer Institute at Zhongshan Hospital (Shanghai, China) and routinely maintained in high-glucose Dulbecco’s modified Eagle’s medium (DMEM) supplemented with 10% fetal bovine serum (FBS), 100 units/mL penicillin, and 100 mg/mL streptomycin. All cells were incubated at 37 °C in a humidified atmosphere containing 5% CO_2_.

### Establishment of knock-down and over-expression cell lines

The coding sequence (CDS) fragments of STYK1 genes were obtained by polymerase chain reaction (PCR) using the following primers: forward, 5′-AAATCTAGAATGGGCATGACACGGATG-3′, 5′-AAAGCGGCCGCTCAAAGCATGCTATAGTTGTAGAAG-3′. The PCR products were then sub-cloned into pCDH-CMV-MCS-EF1-Puro vector (System Biosciences, Mountain View, CA, USA) following the manufacturer protocol. For STYK1 knockdown, short hairpin RNA (shRNA) sequences targeting STYK1 (Sh1: 5′-CCGGGAAGCAGTATGAAGTGATTATCTCGAGATAATCACTTCATACTGCTTCTTTTTTG-3′; Sh2: 5-CCGGGCCCATCTTTCGAGCCAATATCTCGAGATATTGGCTCGAAAGATGGGCTTTTTG-3′; Sh3: 5′-CCGGCAAGTATATCACATCGGAAAGCTCGAGCTTTCCGATGTGATATACTTGTTTTTG-3′) were cloned into pLKO.1-puro vectors (Sigma, St. Louis, MO, USA). The sequences were confirmed by DNA sequencing (Sangon, Shanghai, China).

Lentivirus was packaged in HEK293T cells using X-tremeGENE (Roche, Basel, Switzerland) and the viral DNA was transduced into HCC cell lines. Cells were selected with medium containing 2 μg/ml Puromycin (Sigma) after 72 h of infection.

### Immunohistochemistry and evaluation of staining results

Immunohistochemistry was performed as previously described^34^. Briefly, 4-μm thick tissue sections were used and stained with primary, rabbit polyclonal antibodies against STYK1 (Abcam, Cambridge, MA, USA) at 1:100 dilution and incubated at 4 °C. The stained sections were assessed by 2 independent observers (X.C.Z and W.Z.W.), who were blinded to the clinicopathological information of the patients. STYK1 staining was scored based on the staining intensity and percentage of cell staining, as previously described^34^.

### Colony-formation assay

For colony-formation assays, cells were plated on 6-well plates at a concentration of 800 cells/well and the medium was replaced every 3 days. Cells were fixed and stained with 1% crystal violet after incubating for 2 weeks.

### Cell proliferation, migration and invasion assays

Cell proliferation assay was performed as previously described^34^. Briefly, cells (3 × 10^3^/well) in 100 μl medium were seeded in 96-well plates (Corning, NY, USA). MTT was added to the wells at different time points and cells were incubated at 37 °C for 4 h. Absorbance was measured at 570 nm using a Coulter Counter (Beckman Coulter, Fullerton, CA, USA). Invasion chamber assay with Matrigel (invasion) or without (migration) was performed in triplicate, as previously described^34^. In brief, for migration assay, approximately 3 × 10^4^ cells in serum-free medium were plated into the upper chamber of an 8-μm pore size insert without Matrigel (migration) or with Matrigel (invasion) in the 24-well plate (Corning) and cultured for another 24 h. Cells were fixed in 4% paraformaldehyde and stained with 0.5% crystal violet. The non-migrated/non-invaded cells on the surface of the upper membrane were removed with a cotton swab. The membranes were photographed at 100× magnification under a microscope and the number of migratory/invasive cells was calculated in five randomly selected fields.

### Immunofluorescence

For immunofluorescence microscopy, the cells were seeded on cover slips and incubated with primary antibody against vimentin (1:100, CST, Danvers, MA, USA) followed by incubation with Alexa488-conjugated secondary antibodies (Sigma, St. Louis, MO, USA). Fluorescent staining for vimentin was visualized by confocal laser-scanning microscopy (Fluoview FV1000, Olympus, Japan), using DAPI (Sigma, St. Louis, MO, USA) to counterstain DNA.

### Western blot analysis

Total protein from cells and tissues were lysed in RIPA buffer (CST, Danvers, MA, USA) supplemented with complete protease inhibitor cocktail (Roche, Basel, Switzerland) before use. Equal amount of protein lysates were subjected to 10% sodium dodecyl sulfate-polyacrylamide gel electrophoresis and transferred to polyvinylidene difluoride membranes (Millipore, Billerica, MA, USA). The membrane was blocked in 5% nonfat milk in phosphate-buffered saline supplemented with Tween 20 at room temperature for 1 h and incubated with primary antibody and subsequently with appropriate horseradish peroxidase-conjugated, secondary antibody (1:5000; Pierce, Rockford, IL, USA). Protein bands were visualized by enhanced chemiluminescence detection system (Pierce, Rockford, IL, USA). The primary antibodies against p-AKT (Ser473), AKT, p-ERK (Thr 202/Tyr 204), ERK, E-cadherin, Snail, and GAPDH were obtained from Cell Signaling Technology (Danvers, MA, USA). Fibronectin and Vimentin were obtained from Abcam (Cambrdige, MA, USA). TWIST1 Antibody was obtained from Proteintech (Chicago, IL, USA).

### *In vivo* tumorigenesis and metastasis assays

Nude nu/nu mice (4–6 weeks old) were purchased from the Shanghai Slaccas Animal Center (Shanghai, China). All mice were housed in specific pathogen-free conditions following the guidelines of the Institutional Animal Care and Use Committee of Shanghai Jiao Tong University School of Medicine. To assess the effect of STYK1 on tumorigenicity *in vivo*, 7402 cells (1 × 10^7^ cells) with vector or STYK1 were subcutaneously injected into the left flank of the mice (8 mice/group). Xenograft growth was monitored every week using a caliper. Tumor volume was calculated by the following formula: tumor volume = length/2 × (width/2)^2^. Immunohistochemical staining for STYK1, Vimentin, and E-cadherin was performed on sections from the tumor.

To produce experimental metastases, 7402 cells (1 × 10^6^) with vector or STYK1 were harvested in serum-free DMEM medium and injected into the tail vein of nude mice (10 mice/group). For U0126 and MK-2206 treatment, 7402 cells (1 × 10^6^) stably overexpression STYK1 were randomly injected into the tail vein of nude mice and divided into control and treatment groups. The control group received vehicle (1% DMSO in Saline solution) alone. A dose of 5 mg of the U0126 per kg body weight per day was injected intraperitoneally into mice on days 14 through 21. A dose of 120 mg of the MK-2206 per kg body weight (P.O) per day was administered to mice on days 14 through 21. The mice were sacrificed after 6 weeks and the lungs were surgically excised and stained with H&E. Lung metastatic lesions were evaluated under a dissecting microscope (×200 magnification).

### Statistical analysis

All data are presented as mean value ± standard deviation (SD) from at least 3 independent experiments. Statistical analysis was performed using Student’s *t*-test (two-sided) or analysis of variance. Chi-square (x^2^) or Fisher’s exact test was used to evaluate the associations between the expression of STYK1 and clinicopathological parameters. Cumulative overall and DFS were assessed using Kaplan-Meier’s method and the log-rank test. Statistical analysis was performed using SPSS 16.0 software (SPSS, Inc., Chicago, IL, USA) and p < 0.05 was considered to be statistically significant.

## Additional Information

**How to cite this article**: Wang, Z. *et al*. STYK1 promotes epithelial-mesenchymal transition and tumor metastasis in human hepatocellular carcinoma through MEK/ERK and PI3K/AKT signaling. *Sci. Rep.* **6**, 33205; doi: 10.1038/srep33205 (2016).

## Supplementary Material

Supplementary Information

## Figures and Tables

**Table 1:** Correlation between STYK1 Expression and Clinicopathological Characteristics in 118 HCC Patients.

Variables	STYK1 expression level		p-value
	Low N = 59	High N = 59	
Gender			
Female	12	11	0.816
Male	47	48	
Age, y			
≤51	30	32	0.712
>51	29	27	
HBsAg			
Negative	8	5	0.558*
Positive	51	54	
Liver cirrhosis			
No	8	8	1.000
Yes	51	51	
Tumor size			
≤5cm	52	33	**<0.001**
>5cm	7	26	
Tumor number			
Single	55	48	0.095*
Multiple	4	11	
Tumor envelope			
No	30	31	0.854
Yes	29	28	
TNM stage			
I	43	32	**0.035**
II/III	16	27	
Preoperative AFP(ng/mL)			
≤20	29	20	0.093
>20	30	39	
Vascular invasion			
No	43	32	**0.035**
Yes	16	27	
ALT(units/L)			
≤75	53	56	0.490*
>75	6	3	
Tumor differentiation			
I–II	44	41	0.538
III–IV	15	18	

Abbreviations: ALT, alanine aminotransferase; TNM, tumor-node-metastasis; HBsAg, hepatitis B surface antigen.

*Fisher’s exact tests, and m2 tests for all the other analysis.

**Table 2:** Univariate Analyses of Factors Associated with Overall Survival and Time to Recurrence.

Variable	Overall Survival		Time to Recurrence	
	Hazard ratio (95% CI)	p Value	Hazard ratio (95% CI)	p Value
Sex (male vs. female)	0.791(0.428–1.460)	NS	0.850(0.454–1.427)	NS
Age, y (>52 vs. ≤52)	1.061(0.645–1.745)	NS	1.177(0.734–1.887)	NS
AFP (ng/ml; >20 vs. ≤20)	1.235(0.741–2.060)	NS	1.152(0.713–1.863)	NS
Liver cirrhosis (yes vs. no)	1.627(0.701–3.777)	NS	1.388(0.663–2.902)	NS
ALT (units/L; >75 vs ≤75)	1.530(0.697–3.362)	NS	1.138(0.492–2.630)	NS
Tumor size (cm; >5 vs. ≤5)	1.732(1.028–2.917)	0.039	2.115(1.295–3.454)	0.003
Tumor number (multiple vs. single)	2.040 (1.034–4.026)	0.040	1.936 (1.010–3.708)	0.046
Vascular invasion (yes vs. no)	2.351 (1.423–3.883)	0.001	2.286 (1.416–3.691)	0.001
TNM stage (II/III vs. I)	2.351 (1.423–3.883)	0.001	2.273 (1.422–3.189)	0.001
Tumor differentiation (III/IV vs. I/II)	1.459 (0.855–2.490)	0.166	1.467 (0.887–2.427)	NS
Tumor encapsulation (none vs. complete)	1.170 (0.711–1.926)	0.536	1.268 (0.790–2.034)	NS
STYK1(high vs. low)	2.479 (1.476–4.161)	0.001	2.582 (1.573–2.238)	<0.001

NOTE: Univariate analysis, Cox proportional hazards regression model.

Abbreviation: 95% CI, 95% confidence interval; AFP, alpha-fetoprotein; NS, not significant(Cox proportional hazards regression model); TNM, tumor-node-metastasis.

**Table 3:** Multivariate Analyses of Factors Associated with Overall Survival and Time to Recurrence.

Variable	Overall Survival		Time to Recurrence	
	Hazard ratio (95% CI)	P Value	Hazard ratio (95% CI)	P Value
Tumor size (cm; ≤5 vs. >5)	1.184 (0.680–2.061)	NS	1.445 (0.858–2.435)	0.166
Tumor number (single vs. multiple)	1.267 (0.618–2.599)	NS		
Vascular invasion (no vs. yes)	1.903 (1.121–3.233)	0.017	1.894 (1.161–3.087)	0.010
STYK1 (high vs. low)	1.987 (1.126–3.507)	0.018	2.059 (1.214–3.493)	0.007

NOTE: Multivariate analysis, Cox proportional hazards regression model.

Abbreviation: 95% CI, 95% confidence interval; AFP, alpha-fetoprotein; NS, not significant (Cox proportional hazards regression model); BCLC, Barcelona Clinic Liver Cancer staging system.
