# Supplementary material for: STYK1 promotes epithelial-mesenchymal transition and tumor metastasis in human hepatocellular carcinoma through MEK/ERK and PI3K/AKT signaling
Source: Sci Rep. 2016 Sep 15;6:33205. doi: 10.1038/srep33205 (PMC5024114; doi:10.1038/srep33205)
Supplement: Supplementary Information [file srep33205-s1.pdf]

STYK1 promotes epithelial-mesenchymal transition and tumor metastasis in human hepatocellular carcinoma through MEK/ERK and PI3K/AKT signaling

Zhaowen Wang*, Lei Qu*, Biao Deng, Xing Sun, Shaohan Wu, Jianhua Liao, Junwei Fan†, Zhihai Peng†

Supplementary Figure

Figure 1

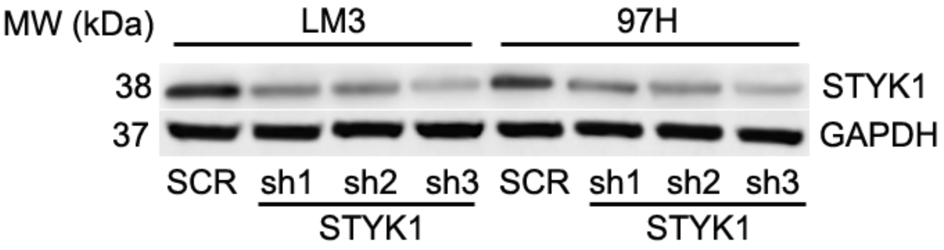

Figure 2

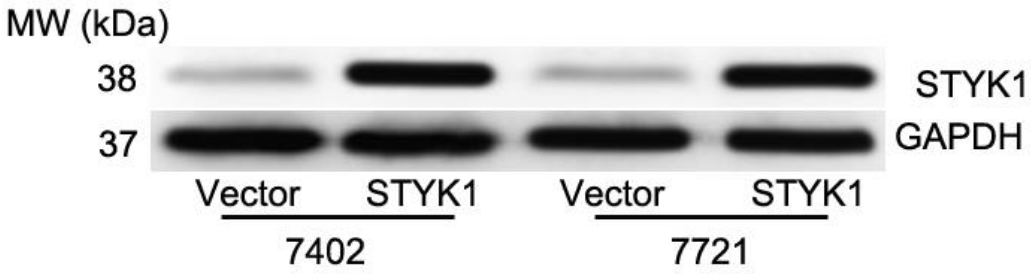

Supplementary Figure legends

Supplementary Figure 1. STYK1 expression in LM3 and 97H HCC cell lines following treatment with shRNA-scramble (SCR) or shRNA-STYK1 (shSTYK1) confirmed reduced expression of STYK1 in cells treated with shSTYK1.

Supplementary Figure 2. STYK1 expression in BEL-7402 and SMMC-7721 HCC cells following transfection of either a control vector (Vector) or STYK1 vector (STYK1) confirmed increased levels of STYK1 in cells transfected with the STYK1 vector.
